# Hospital Contracts and Prices

**Published:** 1910-05-07

**Authors:** 


### Special Departmental Article.

### HOSPITAL CONTRACTS AND PRICES.

### IV. SURGICAL DRESSINGS.

**Contributions and questions on matters affecting this department of an institution are invited.**

Having dealt with drugs, the next most important item under "Surgery and Dispensary" is "Surgical Dressings." One would naturally imagine that there could be little variation in the prices of dressings of the quality known as "Hospital," and that manufacturers, keenly alive to the necessity of producing a good article at a low price for hospital consumption, would manufacture dressings of almost identical quality at practically identical prices; but it is astonishing what variations there are in samples and prices submitted by different firms. The methods of purchase, too, differ considerably in different hospitals. In some cases no contract exists. Hospitals repeat their orders upon the firm which manufactures certain qualities of goods which have been found serviceable, content to pay such prices as the firm may charge or the traveller quote at his periodical call; other hospitals purchase the bulk of their dressings once a month or once a quarter, obtaining tenders from certain selected firms and ordering a monthly or quarterly supply; others make a contract covering a period, ordering the goods from time to time as required.

#### Dressings.

The cotton market of late years has been unfavourable to purchasers. There have been fluctuations, of course, from day to day, week to week, or month to month, but, as a rule, the price of cotton has had an upward tendency, and to-day we should like to see a return of the easy prices of some years ago. Manufacturers of surgical dressings have striven, by improvements in machinery, etc., and by producing articles of slightly inferior quality (but still good enough) to keep the prices of the manufactured articles down, but the fact remains that dressings are more costly than they were in days gone by, in spite of the fact that the improvements in machinery referred to, coupled with the largely increased output consequent upon the greater demand occasioned by the enormous increase in the number of operations, have decreased the cost of manufacture. The practice of aseptic surgery has fortunately reduced the cost of dressings per patient, sterilised and unmedicated gauzes and wools having superseded in great measure the medicated dressings so much in use in days of yore.

#### When should Dressings be Purchased?

Reference was made just now to the different methods of purchasing dressings. It is difficult to say which is the most economical. Possibly a monthly or quarterly quotation, according to the size of the hospital, for a definite supply may be the fairest method of purchase both to the contractor and the hospital in the present uncertain condition of the market, but a half-yearly contract for such quantities as may be required during that period is also a sound arrangement and is useful in administration, as fixed prices for a fairly long period are helpful in calculating the cost of the dressings supplied to the various wards in the regular weekly comparisons, which alone enable a hospital committee to keep a check upon expenditure.

#### Bandages.

Years ago it was customary for all hospitals to make their own bandages, but the introduction of the cheap absorbent variety revolutionised this system and a good many institutions now purchase entirely. We do not think this is wise in the case of calico bandages. An excellent cloth 31 inches wide, quite good enough for the purpose, can be purchased at 2¼d. per yard, and if a really serviceable "winder" is supplied to each surgical ward, the nursing staff can produce a bandage at much less cost than it can be purchased, besides which, experience has proved that the consumption of bandages is much less when they have to be made than when they are purchased ready rolled.

Calico at the price quoted above will wash readily, sometimes two or three times, before the bandage has to be thrown away as past service. A few years ago the same quality of calico could be purchased at 1¾d. or 1⅞d. per yard.

With regard to the open-wove bandage which is so much in evidence nowadays, there is a good deal of difference in the quality used in different hospitals, some authorities being of opinion that the better qualities are most economical as they will stand longer use, others contending that the cheapest kind, namely, fine grey open wove, is good enough for the purpose, and that it should be recognised that this class of bandage will not wash successfully, and should not be used more than once or twice, and that, therefore, the cheapest form obtainable should be purchased. There is a good deal of difference of opinion, too, as to the length of these bandages. At some hospitals it is the practice to use only six-yard lengths, at others five yards are recognised as correct, the opinion being that in the large majority of cases a bandage five yards long is sufficient, and that the other yard is only wasted. Hospitals which are using only six-yard bandages are recommended to try the shorter length. It reduces the cost by one-sixth, and if the longer length is occasionally found to be absolutely necessary, calico or butter-muslin bandages can readily be made by the nursing staff.

Very serviceable five-yard fine grey open-wove bandages can be purchased at the following prices:—

| | |
|---|---|
| 2 inch ... ... ... ... | 5s. 6d per gross |
| 2½ inch ... ... ... ... | 6s. 10d. per gross |
| 3 inch ... ... ... ... | 8s. 1d. per gross |

A good deal of discrimination has to be exercised in the purchase of surgeon's lint. It does not always pay to purchase the cheapest. A very good lint at 1s. 2d. or 1s. 2½d. per lb. has been found to be cheaper in use than one of rather thicker quality at 1s., since it goes much further. This argument, however, does not apply to all dressings, as a hospital which had always used a thick boric lint at 9d. or 9¼d. was persuaded to try a thinner quality of much greater area at 1s. 2d. per lb., expecting to save at least 1d. on each pound used. The idea was that the consumption would be greatly reduced in consequence of the greater area, but in practice, in spite of every care, only a 20 per cent. reduction could be effected, for the lint was thin and extra layers were applied, with the result that the cost of boric lint went up considerably. Needless to say, the experiment was speedily abandoned and the quality at 9*d.* per lb. reverted to.

Difference of opinion also exists as to the price to be paid for white gauze, and in practice it pays to have two qualities. A gauze of fairly open mesh is quite suitable for dressing many cases, but for plugging a better quality with a finer mesh is far preferable; 5s. 1½d. per 100 yards is enough to pay for the great bulk of the gauze, but for plugging purposes one at 6s. 2d. to 6s. 6d. per 100 is more economical and satisfactory. The same remarks apply to cyanide gauze; 6s. 3d. per 100 yards will answer most purposes, but for plugging a gauze at 7s. to 7s. 6d. is found preferable. For ophthalmic work a cyanide gauze at from 12s. to 13s. per 100 yards is preferred by the ophthalmic surgeons, and the consumption being comparatively small, and the risk to eyesight so serious if the eye becomes septic, the use of the higher-priced dressing is to be commended.

A very useful absorbent wool, good enough for all purposes, can be purchased at the present time for 6d. per lb., wood-wool wadding at 7d., grey wool at 4¾d., surgeons' tow 20s. per cwt., iodoform gauze 11s. per 100 yards, guttapercha tissue 4s. 3d. per lb., jacconet 1s. 2d. per yard, oiled paper 8s. per gross.

